# Interpersonal continuity of primary care of veterans with diabetes: a cohort study using electronic health record data

**DOI:** 10.1186/s12875-018-0823-5

**Published:** 2018-07-30

**Authors:** Christine M. Everett, Perri Morgan, Valerie A. Smith, Sandra Woolson, David Edelman, Cristina C. Hendrix, Theodore Berkowitz, Brandolyn White, George L. Jackson

**Affiliations:** 1 Duke University School of Medicine, Physician Assistant Program|, 800 South Duke Street, Durham, NC 27701 USA; 2 Center for Health Services Research in Primary Care, Durham Veterans Affairs Medical Center, Durham, NC USA; 3 Department of Population Health Sciences, Duke University School of Medicine, Durham, NC USA; 4 Division of General Internal Medicine, Duke University School of Medicine, Durham, NC USA; 5 Clinical Health Systems &amp; Analytics Division, Duke University School of Nursing, Durham, NC USA

**Keywords:** Continuity of care, Primary care, Diabetes

## Abstract

**Background:**

Continuity of care is a cornerstone of primary care and is important for patients with chronic diseases such as diabetes. The study objective was to examine patient, provider and contextual factors associated with interpersonal continuity of care (ICoC) among Veteran’s Health Administration (VHA) primary care patients with diabetes.

**Methods:**

This patient-level cohort study (*N* = 656,368) used electronic health record data of adult, pharmaceutically treated patients (96.5% male) with diabetes at national VHA primary care clinics in 2012 and 2013. Each patient was assigned a “home” VHA facility as the primary care clinic most frequently visited, and a primary care provider (PCP) within that home clinic who was most often seen. Patient demographic, medical and social complexity variables, provider type, and clinic contextual variables were utilized. We examined the association of ICoC, measured as maintaining the same PCP across both years, with all variables simultaneously using logistic regression fit with generalized estimating equations.

**Results:**

Among VHA patients with diabetes, 22.3% switched providers between 2012 and 2013. Twelve patient, two provider and two contextual factors were associated with ICoC. Patient characteristics associated with disruptions in ICoC included demographic factors, medical complexity, and social challenges (example: homeless at any time during the year *OR* = 0.79, *CI* = 0.75–0.83). However, disruption in ICoC was most likely experienced by patients whose providers left the clinic (*OR* = 0.09, *CI* = 0.07–0.11). One contextual factor impacting ICoC included NP regulation (most restrictive NP regulation (*OR* = 0.79 *CI* = 0.69–0.97; reference least restrictive regulation).

**Conclusions:**

ICoC is an important mechanism for the delivery of quality primary care to patients with diabetes. By identifying patient, provider, and contextual factors that impact ICoC, this project can inform the development of interventions to improve continuity of chronic illness care.

## Introduction

A core function of primary care is continuity of care (CoC) [1, 2]. CoC is achieved when care is provided as an uninterrupted succession of events and can be achieved through a variety of mechanisms [1]. A cornerstone element of CoC in primary care is interpersonal continuity (ICoC), defined as a longitudinal relationship between a primary care provider (PCP) and patient that involves delivery of preventive care, treatment of multiple illness episodes, and a responsibility for care coordination [3]. ICoC may improve patient-provider communication, the delivery of preventive services and reduce hospitalizations, and may be particularly important for patients with chronic illnesses [4–6]. However, ICoC in the US is lacking, particularly for complex patients. One study suggested that approximately one-third of adults over 65 in the United States (i.e., Medicare beneficiaries) switch their PCP each year [7]. Similarly, staffing approaches which assign patients to a provider-led team do not guarantee ICoC [8].

Patient, organizational, and community factors have been shown to impact CoC. Older, female, more complex, and sicker patients are more likely to achieve CoC, as are patients who do not belong to a racial or ethnic minority [9–12]. Socioeconomic factors including insurance type can be a barrier to ICoC. Healthcare organization size also appears to impact continuity, but data on the direction of the relationship between large organizational size and continuity are contradictory [13–15]. Availability of providers, due to turn-over, training site or other work schedule issues, can lower patient satisfaction and diminish ICoC [15, 16]. In areas with few providers, such as rural settings, continuity may be higher due to fewer choices [17]. To our knowledge however, no study has simultaneously evaluated patient, organizational, and community factors, making it difficult to understand which factors might be the best points of intervention. This paper examines associations between patient, provider, organizational, and community factors with ICoC. Identifying factors that predict ICoC can assist in developing interventions to improve continuity.

## Methods

### Setting

The Veteran’s Health Administration (VHA) is the largest integrated delivery system in the U.S. In 2012, the VHA provided primary care to over 6.33 million patients and had 990 outpatient clinics in 23 regionally defined integrated service networks (VISNs) [18, 19]. Patients who utilize the VHA for care tend to be sicker, older, and have lower incomes than the general population [20]. VHA’s patient-centered medical home model, Patient-Aligned Care Team (PACT), aims to provide strong ICoC within a team-based setting [18, 21]. A panel of approximately 1200 primary care patients are assigned to a PACT, which consists of one PCP (physician, nurse practitioner (NP) or physician assistant (PA)), a registered nurse care manager, a clinical associate (licensed practical nurse, medical assistant or health technologist), and a clerk.

### Data source and sample

**Fig. 1:**
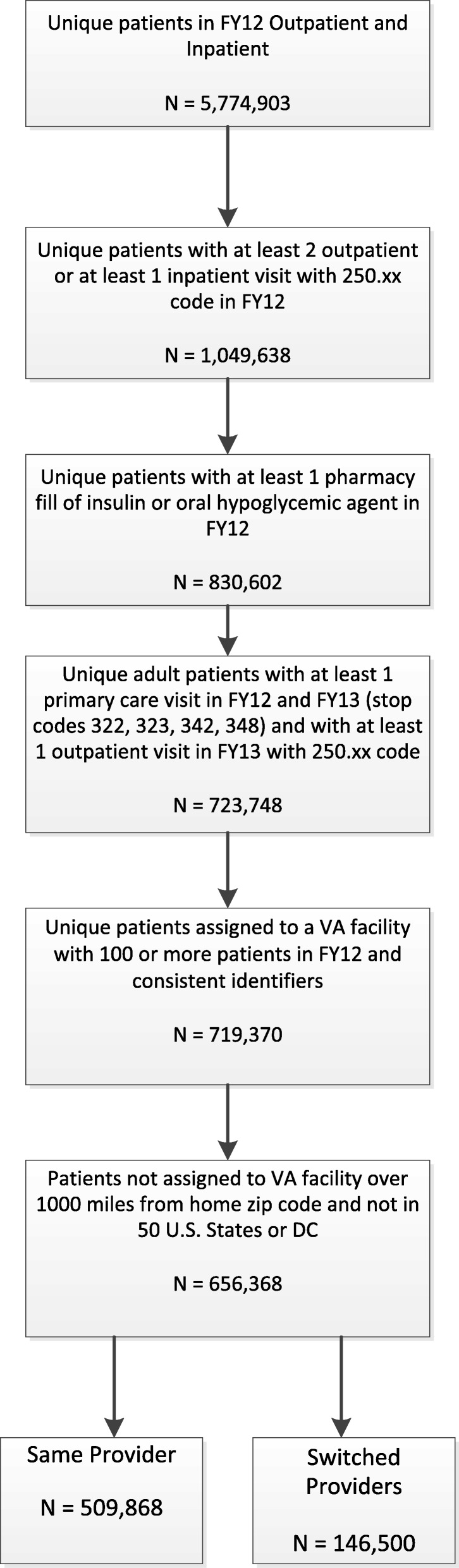
Cohort Construction

This patient-level cohort study used centrally-available national VHA electronic health record data from fiscal years 2012 and 2013 (Fig. 1). The goal of the approach to the study cohort selection was to identify veterans with diabetes that have received a sufficient amount of primary care within the VHA system to impact diabetes outcomes. The sample included adult, pharmaceutically treated veterans with diabetes seen within VHA primary care clinics nationally. Specifically, veterans must have had a diabetes diagnosis (International Classification of Diseases 9th revision (ICD-9) codes 250.xx) associated with at least one VHA inpatient visit and/or at least two a primary care clinic visits (VHA stop codes 322,323,342, and 348) in fiscal year (FY) 2012 (*N* = 1,049,638) and a filled prescription for insulin and/or an oral hyperglycemic agent (VHA drug classes HS501 or HS502) the same year (*N* = 830,602). The combination of ICD-9 and medication criteria was chosen to maximize the likelihood that patients in the dataset have diabetes [22]. Similar algorithms in the VHA have a specificity approaching 100%. Patients were excluded if they did not also have an outpatient visit with a diabetes diagnosis in FY 2013 or were younger than 18. Each patient was assigned a “home” VHA facility as the clinic most frequently visited for primary care in FY 2012. To be retained in the cohort, patients had to have a “home” VHA facility with at least 100 diabetic patients in FY 2012 (*N* = 719,370). This was done to ensure that facilities have experience treating diabetes and provide an extra measure of patient confidentiality. The provider most often visited in the home VHA’s primary care clinic in FY 2012 was considered to be the veteran’s PCP. The same procedure was used to determine home clinic and PCP in FY 2013. We excluded patients whose home VHA facility was &gt; 1000 miles from their home zip code or was not in one of the 50 U.S. states or the District of Columbia, did not have an assigned provider in FY 2012, or had missing information regarding body mass index (*N* = 656,368).

Community data used for explanatory variables was acquired from non-VHA sources. PA scope of practice (SOP) information by state was obtained from a tabulation of recommended key elements assembled by the American Academy of Physician Assistants [23]. NP SOP information was obtained from the 2012 Pearson Report [24]. Both data sources evaluate the extent to which SOP laws in a given state allow PAs or NPs to practice to the level of autonomy considered ideal by each profession’s standards.

### Measures

The outcome of interest was ICoC with a provider between 2012 and 2013. A binary variable was constructed to indicate whether patients switched providers between 2012 and 2013. Patients assigned to the same PCP in 2012 and 2013 were considered to have maintained ICoC. Patients assigned to different PCPs in 2012 and 2013 were considered to have interpersonal discontinuity.

Explanatory variables included patient, provider, organizational and community factors previously demonstrated to be related to ICoC and determined a priori. Patient level demographic factors included age, sex, race, ethnicity, marital status, distance of home address from assigned VHA primary care clinic, and change in home ZIP code between the two years. Patient-level variables suggesting social complexity included homelessness, whether or not the patient was exempt from VHA copayments on the basis of disability or low income, and presence of mental health diagnoses (separate variables for post-traumatic stress disorder (PTSD), mood disorders, substance abuse, and other mental health conditions), and diagnosis of dementia. Patient medical complexity was measured by the Diagnostic Cost Group (DCG) comorbidity measure, originally designed to predict cost of care but validated to measure medical complexity within the VHA population [25, 26]. The algorithm uses demographic and diagnostic information to assign each patient a DCG score, normed so that the average Medicare patient (patients in the U.S. that are 65 years or older, are disabled, or have end stage renal disease) has a score equal to 1 [27]. All patient-level variables were constructed using VHA electronic health record data from FY 2012. Specific categories for each variable can be seen in Table 1.

**Table 1:** Characteristics of VA Patients with Diabetes by Continuity of Care in Primary Care Provider^a^ Type Assigned

Category	Switched Providers *n* = 146,500	Same Provider *n* = 509,868	Total *n* = 656,368
Patient-level factors			
Male	140,638 (96.0)	492,869 (96.7)	633,507 (96.5)
Female	5862 (4.0)	16,999 (3.3)	22,861 (3.5)
Age Group			
Less Than 40	2052 (1.40)	4862 (0.95)	6914 (1.05)
40 to Less Than 65	80,264 (54.8)	261,109 (51.2)	341,373 (52.0)
65 to Less Than 80	52,682 (36.0)	196,840 (38.6)	249,522 (38.0)
80 and Over	11,502 (7.85)	47,057 (9.23)	58,559 (8.92)
Race			
White	103,597 (70.7)	363,036 (71.2)	466,633 (71.1)
American Indian	1289 (0.88)	3678 (0.72)	4967 (0.76)
Asian	918 (0.63)	2832 (0.56)	3750 (0.57)
Black	26,975 (18.4)	91,991 (18.0)	118,966 (18.1)
Native Hawaiian	1599 (1.09)	5596 (1.10)	7195 (1.10)
Unknown or Missing	12,122 (8.27)	42,735 (8.38)	54,857 (8.36)
Hispanic	8561 (5.84)	24,157 (4.74)	32,718 (4.98)
Marital Status			
Currently Married	84,329 (57.6)	308,274 (60.5)	392,603 (59.8)
Never Married	17,016 (11.6)	54,421 (10.7)	71,437 (10.9)
Previously Married	44,772 (30.6)	145,689 (28.6)	190,461 (29.0)
Unknown Marital Status	383 (0.26)	1484 (0.29)	1867 (0.28)
Homeless at Any Time During Year	4549 (3.11)	8711 (1.71)	13,260 (2.02)
Copay Status			
No Copay Due to Disability	81,123 (55.4)	278,359 (54.6)	359,482 (54.8)
No Copay Due to Low Income	40,864 (27.9)	133,272 (26.1)	174,136 (26.5)
Must Pay Copay	22,502 (15.4)	91,157 (17.9)	113,659 (17.3)
Copay Status Unknown	2011 (1.37)	7080 (1.39)	9091 (1.39)
Mental Health Diagnoses			
Mood Disorder	39,135 (26.7)	121,321 (23.8)	160,456 (24.4)
Post-Traumatic Stress Disorder	22,683 (15.5)	72,896 (14.3)	95,579 (14.6)
Dementia	4957 (3.38)	15,236 (2.99)	20,193 (3.08)
Substance Abuse	13,689 (9.34)	38,160 (7.48)	51,849 (7.90)
Other Mental Health Diagnosis	8782 (5.99)	30,004 (5.88)	38,786 (5.91)
Diagnostic Cost Group (DCG) Score Category			
Less Than or Equal to 0.5	69,654 (47.5)	260,010 (51.0)	329,664 (50.2)
Greater Than 0.5 to 1	25,123 (17.1)	86,700 (17.0)	111,823 (17.0)
Greater Than 1 to 1.5	19,313 (13.2)	64,932 (12.7)	84,245 (12.8)
Greater Than 1.5 to 2	11,228 (7.66)	35,519 (6.97)	46,747 (7.12)
Greater Than 2	21,182 (14.5)	62,707 (12.3)	83,889 (12.8)
Distance from VHA Primary Care Clinic			
Less Than 5 Miles	31,941 (21.8)	119,774 (23.5)	151,715 (23.1)
5 to Less Than 25 Miles	71,412 (48.7)	265,553 (52.1)	336,965 (51.3)
25 to Less Than 50 Miles	24,976 (17.0)	83,394 (16.4)	108,370 (16.5)
50 Miles and Over	17,018 (11.6)	37,448 (7.34)	54,466 (8.30)
Missing	1153 (0.79)	3699 (0.73)	4852 (0.74)
Baseline BMI			
Less Than 18.5	363 (0.25)	1152 (0.23)	1515 (0.23)
18.5 to Less Than 25	13,566 (9.26)	47,100 (9.24)	60,666 (9.24)
25 to Less Than 30	42,293 (28.9)	149,814 (29.4)	192,107 (29.3)
30 to Less Than 35	46,015 (31.4)	160,415 (31.5)	206,430 (31.5)
35 and Above	44,263 (30.2)	151,387 (29.7)	195,650 (29.8)
Number of PC Visits			
1 PC Visit	39,830 (27.2)	103,854 (20.4)	143,684 (21.9)
2 PC Visits	38,382 (26.2)	192,864 (37.8)	231,246 (35.2)
3 PC Visits	32,305 (22.1)	104,846 (20.6)	137,151 (20.9)
4 or More PC Visits	35,983 (24.6)	108,304 (21.2)	144,287 (22.0)
Pharmacy Fill of Insulin	65,784 (44.9)	216,765 (42.5)	282,549 (43.0)
Patient Had Same Zip Code in FY12 and FY13	127,158 (86.8)	478,420 (93.8)	605,578 (92.3)
Provider-level factors			
Assigned Provider Type in FY12			
Physician	104,395 (71.3)	394,959 (77.5)	499,354 (76.1)
Nurse Practitioner	25,616 (17.5)	79,588 (15.6)	105,204 (16.0)
Physician Assistant	10,185 (6.95)	30,798 (6.04)	40,983 (6.24)
Physician Resident	6304 (4.30)	4523 (0.89)	10,827 (1.65)
Assigned Provider Type in FY13			
Physician	87,706 (59.9)	395,286 (77.5)	482,992 (73.6)
Nurse Practitioner	20,564 (14.0)	79,464 (15.6)	100,028 (15.2)
Physician Assistant	7178 (4.90)	30,716 (6.02)	37,894 (5.77)
Physician Resident	5051 (3.45)	4402 (0.86)	9453 (1.44)
Unable to Assign	26,001 (17.7)		26,001 (3.96)
Provider Turnover from Station	24,512 (16.7)	8746 (1.72)	33,258 (5.07)
Facility-level factors			
Endocrinology Referral Capacity^b^	70,437 (48.1)	238,805 (46.8)	309,242 (47.1)
Rural Urban Commuting Area Status			
Metropolitan Area Core	106,700 (72.8)	377,339 (74.0)	484,039 (73.7)
Metropolitan Area Core - Remaining Levels	16,423 (11.2)	62,616 (12.3)	79,039 (12.0)
Micropolitan Area Core	18,003 (12.3)	53,501 (10.5)	71,504 (10.9)
Small Town or Rural	5374 (3.67)	16,412 (3.22)	21,786 (3.32)
State-level factors			
Percent of Primary Care Physicians Who Work With NPs/PAs			
Lowest Tertile	78,653 (53.7)	284,992 (55.9)	363,645 (55.4)
Middle Tertile	38,373 (26.2)	133,094 (26.1)	171,467 (26.1)
Highest Tertile	29,474 (20.1)	91,782 (18.0)	121,256 (18.5)
Nurse Practitioner Scope of Practice Regulations			
Least Restrictive	23,738 (16.2)	69,747 (13.7)	93,485 (14.2)
Moderately Restrictive	22,697 (15.5)	79,056 (15.5)	101,753 (15.5)
Most Restrictive	100,065 (68.3)	361,065 (70.8)	461,130 (70.3)
Physician Assistant Scope of Practice Regulations			
Least Restrictive	20,774 (14.2)	69,521 (13.6)	90,295 (13.8)
Moderately Restrictive	36,532 (24.9)	113,202 (22.2)	149,734 (22.8)
Most Restrictive	89,194 (60.9)	327,145 (64.2)	416,339 (63.4)
VISN-LEVEL factors			
Region			
Northeast	16,522 (11.3)	75,920 (14.9)	92,442 (14.1)
West	32,865 (22.4)	91,014 (17.9)	123,879 (18.9)
Midwest	31,983 (21.8)	116,774 (22.9)	148,757 (22.7)
South	65,130 (44.5)	226,160 (44.4)	291,290 (44.4)

Data for patient-level variables are from the Veterans Administration electronic health record files. Other data sources are described in the Methods section

^a^Primary care provider (PCP) is assigned as the physician, NP, or PA seen most during FY 2012 and 2013

^b^Endocrinology referral capacity is defined as either present (endocrinology or other diabetes mellitus specialty clinics provided 500 or more visits to cohort patients in FY12) or absent (fewer than 500 visits to cohort patients)

Two provider-level variables were measured and assigned to each patient based on their assigned provider in 2012. Primary care provider type was represented by a categorical variable (staff physician, resident, NP, PA). A variable indicating provider turnover was also created. Since VHA providers can commonly take up to 3 months off work for health or professional issues, provider turn-over was considered to have occurred if a provider did not provide any visits within the clinic in a consecutive 4 month period. Primary care provider assignment to a clinic in FY 2012 was compared to visits performed at the same assigned station in FY 2013. If there was a 4 month or longer period in which a provider did not perform a single primary care visit at the station in FY 2013 then the provider was flagged as having left the clinic. If a PCP performed no visits in the final 4 months of year, they were also considered to have left the clinic (turn-over = 1).

Organizational level variables were assessed for each facility. The value for each facility variable was calculated based on the patient clinic assignment in 2012. Region of the country was categorized as West, Midwest, Northeast, South and rurality was indicated by rural urban commuting area (RUCA) of the facility location (metropolitan area core, micropolitan, other metropolitan area, small town or rural). If a facility had 500 or more clinic stops (visits) for 305 or 306 (VHA codes for an endocrinology visit) in FY 2012 then the facility was defined as having endocrinology referral capacity, designated with an indicator variable.

State level variables were identified based on the state in which the facility is located. State variables include the percent of primary care physicians who work with NPs or PAs within the state (lowest, middle and highest tertile), NP and PA SOP regulations (least restrictive, moderately restrictive, and most restrictive). The value for each state variable was based on the state of the patient’s home clinic assignment in 2012.

### Analysis

All analyses were conducted using SAS® 9.4 [28] and SAS Enterprise Guide 7.1 [29]. Descriptive statistics were calculated for all variables at the patient-level. The association between experiencing ICoC with a PCP and patient, provider, facility, and state contextual variables was evaluated using logistic regression fit with generalized estimating equations and an exchangeable correlation structure to account for clustering within facilities. Covariates were specified a priori and assessed for multicollinearity prior to being entered into the model. All analyses set statistical significance at *p* &lt; 0.05.

### IRB approval

This work was reviewed and approved by the Internal Review Board (IRB) of the Durham Veterans Affairs Health Care System. Reflecting that this is a secondary data study, the IRB approved a waiver of informed consent for this study.

## Results

### Patient characteristics

Approximately 18% (*N* = 1,049,638) of VHA patients had pharmaceutically treated diabetes mellitus (Fig. 1). Among the patients with diabetes that could be assigned to a PCP (*N* = 656,368) in 2012, 76% were assigned to an attending physician, 16% were assigned to an NP, 6.2% were assigned to a PA, and 1.7% were assigned to a resident physician. Approximately 22.3% of VHA patients with diabetes switched providers between 2012 and 2013. Switching providers occurred in 20.9% of patients assigned to physicians, 24.9% of patients assigned to PAs, 24.3% of patients assigned to NPs, and 58.2% of patients assigned to resident physicians.

Characteristics of the study population were similar to the general VHA population (Table 1). Patients with diabetes were predominantly older (mean age = 64.9 [standard deviation (SD) = 10.1]; less than 2% were under 40 years old), and were predominantly non-Hispanic (95%), white (71%), and male (97%). The study population was medically complex with approximately 20% of patients having at least 50% higher utilization (being at least 50% more complex) than the average Medicare patient (i.e., the oldest and sickest patients in the U. S.) based on the DCG score and high rates of mental health disorders (mood disorders 24%, post-traumatic stress disorder 15%). Social complexity is also prevalent, with 55% of the sample having no copay due to disability, 27% having no copay due to income, and 2% experiencing homelessness during fiscal year 2012.

#### Facility and contextual characteristics

Primary care was delivered to the study sample in 831 facilities in all regions of the United States (22% in the West, 25% in the Midwest, 19% in the Northeast, and 34% in the South), with 53% located in metropolitan areas (Table 1). The mean number of providers with diabetes patients in the cohort per facility was 12.9 (*SD* = 20.2). The facilities had a range of staffing mixes, with the average percent of attending physicians, NPs, PAs, and residents at 74, 15, 6, and 1.4% respectively. Approximately 46% of the primary care facilities had provider turn-over between FY 2012 and FY 2013. Facilities were located in states with a range of SOP settings for NPs and PAs. Approximately 27% of facilities were in states with the most restrictive NP SOP regulation and 64% of facilities were in states with the most restrictive PA SOP regulation.

#### Factors associated with interpersonal continuity with a primary care provider

After adjustment for all other factors in the model, patient, provider, facility and contextual factors were associated with interpersonal continuity (Table 2). Demographic factors associated with increased odds of ICoC include male gender (*OR* = 1.14 *CI* 1.05–1.23), increasing age (age 40 to &lt; 65 *OR* = 1.23 *CI* 1.16–1.30; age 65 to &lt; 80 *OR* = 1.30 *CI* 1.23–1.38; age &gt; 80 *OR* = 1.35 *CI* 1.27–1.44; reference age &lt; 40), and living in the same zip code throughout both years (*OR* = 2.20 CI 2.12–2.28; reference changing zip codes) Demographic factors associated with slightly decreased odds of ICoC include copay status (no copay due to disability *OR* = 0.98 *CI* = 0.96–0.99; no copay due to low income *OR* = 0.95 CI 0.94–0.97; copay status unknown *OR* = 0.93 *CI* = 0.88–0.99; reference must pay copay), Hispanic ethnicity (*OR* = 0.96 *CI* = 0.93–0.99), marital status (never married *OR* = 0.98 *CI* = 0.96–1.00; previously married *OR* = 0.97 *CI* = 0.96–0.98; reference currently married) and distance from VHA primary care clinic (5 to &lt; 25 miles *OR* = 0.97 CI 0.95–0.99; 25 to &lt; 50 miles *OR* = 0.85 *CI* = 0.83–0.88; &gt; 50 miles *OR* = 0.62 *CI* = 0.59–0.65; reference &lt; 5 miles).

**Table 2:** Odds Ratios and 95% CI for Predicting Continuity of Care

Effect and Level	Odds Ratio	95% CI	*P*-Value
Patient-level factors			
Male	1.14	(1.05,1.23)	0.002
Age Group			
Less Than 40	Reference	Reference	
40 to Less Than 65	1.23	(1.16,1.30)	&lt;.001
65 to Less Than 80	1.30	(1.23,1.38)	&lt;.001
0 and Up	1.35	(1.27,1.44)	&lt;.001
Race			
White	Reference	Reference	
American Indian	0.98	(0.91,1.05)	0.549
Asian	0.95	(0.88,1.04)	0.266
Black	1.02	(0.99,1.04)	0.124
Native Hawaiian	1.00	(0.95,1.06)	0.956
Unknown or Missing	1.02	(0.99,1.04)	0.188
Hispanic	0.96	(0.93,0.99)	0.011
Marital Status			
Currently Married	Reference	Reference	
Never Married	0.98	(0.96,1.00)	0.038
Previously Married	0.97	(0.96,0.98)	&lt;.001
Unknown Marital Status	0.98	(0.90,1.08)	0.687
Homeless at Any Time During Year	0.79	(0.75,0.83)	&lt;.001
Copay Status			
Must Pay Copay	Reference	Reference	
No Copay Due to Disability	0.98	(0.96,0.99)	0.010
No Copay Due to Low Income	0.95	(0.94,0.97)	&lt;.001
Copay Status Unknown	0.93	(0.88,0.99)	0.013
Mental Health Diagnoses			
Mood Disorder	0.97	(0.95,0.98)	&lt;.001
Substance Abuse	0.94	(0.92,0.96)	&lt;.001
PTSD	0.99	(0.97,1.01)	0.399
Dementia	0.94	(0.90,0.97)	&lt;.001
Other Mental Health Diagnosis	0.99	(0.96,1.02)	0.650
Diagnostic Cost Group (DCG) Score Category			
Less Than or Equal to 0.5	Reference	Reference	
Greater Than 0.5 to 1	0.95	(0.93,0.97)	&lt;.001
Greater Than 1 to 1.5	0.93	(0.91,0.95)	&lt;.001
Greater Than 1.5 to 2	0.91	(0.89,0.94)	&lt;.001
Greater Than 2	0.89	(0.86,0.91)	&lt;.001
Distance From VHA Primary Care Clinic			
Less Than 5 Miles	Reference	Reference	
5 to Less Than 25 Miles	0.97	(0.95,0.99)	0.001
25 to Less Than 50 Miles	0.85	(0.83,0.88)	&lt;.001
50 Miles or Greater	0.62	(0.59,0.65)	&lt;.001
Missing	1.15	(1.01,1.30)	0.034
Baseline BMI			
Less Than 18.5	Reference	Reference	
18.5 to Less Than 25	0.97	(0.86,1.09)	0.611
25 to Less Than 30	0.98	(0.87,1.10)	0.723
30 to Less Than 35	0.97	(0.86,1.09)	0.600
Greater Than or Equal to 35	0.97	(0.86,1.09)	0.599
Number of PC Visits in FY12			
1 Visit	Reference	Reference	
2 Visits	1.78	(1.72,1.85)	&lt;.001
3 Visits	1.25	(1.21,1.30)	&lt;.001
4 or More Visits	1.23	(1.18,1.29)	&lt;.001
Patient Had Same Zip Code	2.20	(2.12,2.28)	&lt;.001
Provider-level factors			
Assigned Provider Type in FY12^a^			
Physician	Reference	Reference	
Nurse Practitioner	0.87	(0.78,0.97)	0.011
Physician Assistant	0.86	(0.73,1.02)	0.081
Physician Resident	0.18	(0.15,0.21)	&lt;.001
Provider Turnover	0.09	(0.07,0.11)	&lt;.001
Facility-level factors			
Presence of Endocrinology at Facility^b^	1.06	(0.89,1.25)	0.514
Rural Urban Commuting Area Status			
Metropolitan Area Core	Reference	Reference	
Other Metropolitan Area	1.07	(0.89,1.28)	0.467
Micropolitan	0.98	(0.82,1.16)	0.778
Small Town or Rural	0.81	(0.59,1.11)	0.193
State-level factors			
Percent of Primary Care Physicians Who Work With NPs/PA			
Lowest Tertile	Reference	Reference	
Middle Tertile	0.88	(0.72,1.07)	0.204
Highest Tertile	0.95	(0.74,1.22)	0.684
Nurse Practitioner Scope of Practice Regulations			
Least Restrictive	Reference	Reference	
Moderately Restrictive	0.92	(0.71,1.20)	0.545
Most Restrictive	0.79	(0.64,0.97)	0.027
Physician Assistant Scope of Practice Regulations			
Least Restrictive	Reference	Reference	
Moderately Restrictive	1.06	(0.83,1.36)	0.629
Most Restrictive	1.17	(0.93,1.46)	0.183
Visn-level factors			
Region			
Northeast	Reference	Reference	
West	0.85	(0.69,1.03)	0.100
Midwest	1.34	(1.06,1.70)	0.016
South	0.87	(0.73,1.04)	0.127

Data for patient-level variables are from the Veterans Administration electronic health record files. Other data sources are described in the Methods section

^a^Primary care provider (PCP) is assigned as the physician, NP, or PA seen most during FY 2012 and 2013

^b^Endocrinology referral capacity is defined as either present (endocrinology or other diabetes mellitus specialty clinics provided 500 or more visits to cohort patients in FY12) or absent (fewer than 500 visits to cohort patients)

Patients with worse overall health status had modestly lower odds of ICoC from year to year. Higher DCG score categories in FY 2012 (i.e., complex patients with greater healthcare utilization) had lower odds of continuity (DCG 0.5–1.0 *OR* = 0.95 *CI* = 0.93–0.97; 1.0–1.5 *OR* = 0.93 *CI* = 0.91–0.95; 1.5–2.0 *OR* = 0.91 *CI* = 0.89–0.94; &gt; 2.0 *OR* = 0.89 *CI* = 0.86–0.91; reference &lt; 0.5). This is also seen for patients with a mental health diagnosis (mood disorder *OR* = 0.97 CI 0.95–0.98; substance abuse *OR* = 0.94 *CI* = 0.92–0.96; dementia *OR* = 0.94 *CI* = 0.90–0.97). Finally, a greater number of primary care visits in FY 2012 was associated with increased odds of ICoC (2 visits *OR* = 1.78 *CI* = 1.72–1.85; 3 visits *OR* = 1.25 *CI* = 1.21–1.30; 4+ visits *OR* = 1.23 *CI* = 1.18–1.29; reference = 1 visit).

Both provider variables were associated with decreased odds of ICoC. Patients who had a provider that left the clinic had a highly significant and large decrease in odds of ICoC (*OR* = 0.09, CI-0.07-0.11) when compared to patients assigned to providers that stayed in the clinic. Compared to patients assigned to an attending physician, patients with NPs (*OR* = 0.87 *CI* = 0.78–0.97) and resident physicians (*OR* = 0.18 *CI* = 0.15–0.21) as usual providers had decreased odds of ICoC.

Facility and contextual variables were also associated with ICoC. Compared to patients receiving primary care services in the Northeast, patients in the Midwest had increased odds of ICoC (*OR* = 1.34 *CI* = 1.06–1.70). Compared to patients that received primary care services at VHA clinics in states with the least restrictive NP SOP regulations, patients that received care in states with the most restrictive NP regulations had decreased odds of ICoC (*OR* = 0.79 *CI* = 0.69–0.97).

## Discussion

Approximately 22% of VHA patients with diabetes experienced disruption in ICoC between 2012 and 2013, which is less than the one-third of Medicare patients in the US previously reported to change primary care providers from year to year [7]. Findings suggest that, when assessed simultaneously across the VHA, patient, provider, and contextual factors are associated with ICoC for patients with diabetes. Patients with social and access challenges are less likely to experience ICoC. Similarly, region of the country and NP scope of practice regulation also impacted the likelihood of experiencing ICoC. However, the biggest impact appears to be associated with a provider-level variable: provider turn-over.

Even after adjustment for provider and contextual factors, patients with diabetes with medical complexity, social and access challenges were less likely to experience ICoC. Consistent with existing literature, patients with diabetes with sociodemographic factors including Hispanic ethnicity, female gender, marital status, low income, and living a greater distance from the clinic were less likely to experience ICoC [11, 12, 30]. Similarly, patients with greater medical complexity and mental health issues were less likely to experience ICoC. However, effect sizes for the aforementioned variables were small; the patient factors with the largest effect were patient living in the same zip code, age and primary care utilization. Older age groups and those with more primary care visits were more likely to experience ICoC. Since this effect was seen after controlling for patient complexity, this finding is less likely due to the medical needs of the patients and more likely due to patient preference [9, 10].

Contextual factors that impacted patient ICoC include region of the country and NP SOP regulations. Patients receiving care in the Midwestern US were more likely than those receiving care in the Northeast to experience ICoC. Several potential explanations exist. It could relate to regional differences in healthcare utilization patterns [31]. However, it is also likely that VHA expansion can explain at least some of the effect. For example, the VHA had 152,000 more patients in 2013 than 2012 [19]. Similarly, patients that lived in states with the most restrictive NP scope of practice regulations were less likely to experience ICoC. While the mechanism is unknown, this may be due to greater interdependence between NPs and physicians, resulting in more patient-sharing which appears like provider switching using our methodology.

Provider type and provider turn-over also appeared to impact ICoC for patients with diabetes. Compared to patients with attending physicians as usual providers, patients with physician residents and NPs were less likely to experience ICoC. The magnitude of effect was greatest with residents. Given the nature of physician training, in which trainees frequently rotate through other learning experiences, it is not unexpected that patients that receive the majority of their care from a resident physician would experience interpersonal discontinuity. However, the proportion of patients that receive care from resident physicians is small (1.7%). Patients with NPs as PCPs had only slightly lower odds of experiencing ICoC than those with physicians. This could be due to a variety of factors. It could be that patients with diabetes are dissatisfied with the care received from NPs and elect to switch providers. This explanation seems unlikely, given that existing literature suggests that satisfaction with and quality of care delivered by primary care NPs is as good as or better than satisfaction with physicians [32–35]. Alternatively, it could be due to policy changes regarding primary care NP roles within the VHA or to patient reassignent [18, 36–38]. The impact of provider turn-over appeared to have a far greater impact on continuity of care than provider type. Provider turn-over within the VHA has been high and increased after PACT implementation [37, 39]. Our data suggest that approximately 46% of primary care clinics had provider turn-over between 2012 and 2013, suggesting that turn-over has the potential to directly or indirectly impact a significant portion of VHA patients with diabetes.

This study has notable strengths and limitations. Unlike previous studies, our study has simultaneously evaluated patient, provider and contextual factors that can influence ICoC. This approach allows for a better understanding of the contribution of each factor to continuity. Unlike many commonly used data sources, our data also allows for accurate attribution of the performing provider of care for each patient with diabetes [40].

Several limitations must also be recognized. There is potential for misclassification of assignment to usual provider. Our methodology utilized only face-to-face visits with patients. Since PACT implementation, there has been a significant increase in the number of electronic encounters with patients such as phone and electronic communications, and could potentially disproportionately impact those patients living further away from their VHA facility [21]. However, many of these excluded encounters are provided by nurses other than NPs and other professionals that are not acting in usual provider roles [21]. Similarly, it is possible that patients could have had a minority of their primary care visits in 2013 with their PCP from 2012 even if they were assigned to a new PCP in 2013. However, including these patients in the “discontinuity” category would bias the findings toward the null; making any estimates provided conservative estimates of effect. Finally, despite the fact that our models included a large variety of variables that have been shown to predict ICoC, there is the potential for unmeasured confounding. For example, patient preferences were not addressed and may have provided some additional clarification [41].

## Conclusion

ICoC of care is an important mechanism for the delivery of high quality primary care to patients with chronic illness such as diabetes. This paper contributes critical knowledge, by identifying patient, provider, and contextual factors that impact ICoC. Identification of factors associated with ICoC can assist with the development of interventions to improve chronic illness care. We found that patients with diabetes who are younger, with medical complexity, social and access challenges, are less likely to experience ICoC. This suggests that interventions to improve continuity may need to target these patients. Additionally, provider and contextual factors, especially provider turn-over, are reducing ICoC. These factors are likely sensitive to organizational policies, suggesting that VHA and other healthcare system administrators may wish to re-evaluate policies that impact NP roles and provider turn-over.

## Abbreviations

CI: Confidence interval
CoC: Continuity of Care
DCG: Diagnostic cost group
FY: Fiscal year
ICD-9: International Classification of Diseases 9th revision
ICoC: Interpersonal continuity of care
NP: Nurse practitioner
OR: Odds ratio
PA: Physician assistant
PACT: Patient-Aligned Care Team
PCP: Primary care provider
PTSD: Post-traumatic stress disorder
RUCA: Rural urban commuting area
SOP: Scope of practice
VHA: Veteran’s Health Administration
